# MNSs Blood Group Glycophorin Variants in Taiwan: A Genotype-Serotype Correlation Study of ‘Mi^a^’ and St^a^ with Report of Two New Alleles for St^a^

**DOI:** 10.1371/journal.pone.0098166

**Published:** 2014-05-23

**Authors:** Tai-Di Chen, Ding-Ping Chen, Wei-Ting Wang, Chien-Feng Sun

**Affiliations:** 1 Department of Anatomic Pathology, Chang Gung Memorial Hospital, Taoyuan County, Taiwan; 2 Department of Laboratory Medicine, Chang Gung Memorial Hospital, Taoyuan County, Taiwan; 3 Department of Medical Biotechnology and Laboratory Science, Chang Gung University, Taoyuan County, Taiwan; 4 Department of Pathology, School of Medicine, Chang Gung University, Taoyuan County, Taiwan; German Red Cross Blood Service Frankfurt, Germany

## Abstract

**Background:**

Glycophorin variants of the MNSs blood group are important in Taiwan. For more than 20 years, screening for the most frequent irregular antibody, anti-‘Mi^a^’, has been conducted by using ‘Mi^a^’(+) RBCs, with a significant success. However, the sensitivity and the specificity of this screening strategy have never been validated, and the true incidences of different glycophorin variants in Taiwan have been in controversy. Also, the significance of another less frequent and usually separately reported variant, St^a^, has never been evaluated.

**Methodology/Principal Findings:**

We ran a population-based screening (from unselected patients in our hospital) for MNSs blood group glycophorin variants by PCR-sequencing method. GP.Mur (Mil.III) was confirmed by sequence from 57 out of 1027 samples (5.6%), and there was no other Miltenberger subtype glycophorin variant found. Glycophorin variant St^a^ was found from 35 out of 1027 samples (3.4%). In contrast to anti-‘Mi^a^’, which is the most frequently identified irregular antibody in Taiwan, the prevalence of anti-St^a^ was only 0.13% as determined by serologic method. In addition, two new alleles for St^a^ were found and reported.

**Conclusion/Significance:**

We confirm the long-standing assumption that GP.Mur is the only prevalent Miltenberger subtype in Taiwan. The current anti-‘Mi^a^’ screening method used in Taiwan, although neither sensitive nor specific, is still a suitable practice. Although St^a^ antigen has a high prevalence in Taiwan, routine screening for anti-St^a^ is not warranted based on current evidence.

## Introduction

MNSs blood group antigens are carried on two major membrane sialoglycoprotein, glycophorin A (GPA) and glycophorin B (GPB). They are encoded by glycophorin A gene (*GYPA*) and glycophorin B gene (*GYPB*), respectively. Due to the similarity of *GYPA* and *GYPB*, unequal crossing-over or gene conversion can happen, and results in hybrid genes encoding structural variant glycophorins [1]. These glycophorin variants are initially recognized by serologic methods and named as Miltenberger subsystem. Later, the Miltenberger nomenclature was replaced by GP. name terminology with the name denoting the first propositus [2]. Previously known as Miltenberger subtype III (Mi.III), GP.Mur is the glycophorin product of a hybrid *GYP(B-A-B)* gene on the red blood cell (RBC) membrane. GP.Mur was reported to have a mean frequency of 7.3% among Taiwan population, and is an important blood group antigen in Taiwan [3]–[6].

In Caucasian, most of the Miltenberger antigens and antibodies are rare. However, antibodies against GP.Mur are the most frequently detected irregular antibodies in Taiwan blood banks. The sera against GP.Mur are mixtures composed of different antibodies, namely anti-Mia, anti-Mur, anti-MUT, anti-Hil, and anti-MINY. Since these polyspecific antibodies are difficult to be separately isolated and specifically identified individually, they have been collectively called anti-‘Mi^a^’ in Taiwan. Taiwan Society of Blood Transfusion has issued a guideline of including ‘Mi^a^’(+) cells in the antibody screening panel, and this practice has been conducted for more than 20 years. However, the sensitivity and specificity of this screening strategy have never been validated. Shih et al. used anti-‘Mi^a^’ sera to test 200 individuals' RBCs and found serologically positive in 19 cases [7]. However, only 9 out of those 19 individuals were found to be GP.Mur genotypically positive. This finding indicates that the currently detected anti-‘Mi^a^’ sera might be heterogeneous, and might be composed of unknown antibodies against antigen other than those belonging to Gp.Mur.

St^a^ antigen, first described by Cleghorn in 1962 [8], is another glycophorin variant resulted from hybrid gene *GYP(B-A)* with a single unequal crossing over at intron 3 of *GYPA* and *GYPB*. It contains the N-terminal part of *GYPB* and the C-terminal part of *GYPA* [1]. Currently, four distinct alleles for St^a^ (type A to D) are identified, differing in the location of crossing-over points [9].

St^a^ antigen is rarely found in Caucasians, but is significantly highly prevalent (6.36%) in Japan [10]. Reports from other Asia countries such as China and Taiwan also show high frequencies, ranging from 1.63% to 5.2% [7], [11], [12]. Antibodies to St^a^ antigen had been reported [8], [10]. St^a^ antigen, like the prevalent GP.Mur antigen in Asian population, is the product of hybrid glycophorin gene, thus may have similarly strong antigenicity as GP.Mur. However, due to the rarity of reports and the lack of large scale studies on either St^a^ antigen or anti-St^a^ in the population, the significance of St^a^ in transfusion medicine, especially in Asian population, has not be determined.

In this study, we want to address the following issues, 1) the genotypic frequencies of each glycophorin variants including Miltenberger subtypes and St^a^ in Taiwan, 2) to validate efficacy of current anti-‘Mi^a^’ screening method, and 3) the significance of St^a^ antigen in Taiwan transfusion practice.

## Materials and Methods

### Ethics Statement

This study was approved by institution review board (IRB) of Chang Gung Memorial Hospital (approved number: 99-3397A3). All the participants provided their written informed consent to participate in this study.

### The Frequencies of Glycophorin Variants Including Miltenberger Subtypes and St^a^

One thousand and twenty seven (1027) patient's whole blood samples were obtained from the blood bank of Chang Gung Memorial Hospital. DNA concentrates were extracted from whole blood samples using QIAmp Extraction Kit (Valencia, CA, USA). Genomic DNA extracted from the whole blood samples (0.1 ug/reaction) was thawed on ice and added to a reaction mixture containing 20 pmol of each primer, 200 uM of each dNTP, 2.0 units of Taq polymerase, and 50 uL of reaction buffer (100 ug/uL gelatin, 1.5 mMMgCl2, 10 mMTris-HCl[pH8.4], and 50 mMKCl). Genomic DNA was amplified by polymerase chain reaction (PCR) using different sets of primers and programs listed in Table 1, Table 2, and Table 3. For *GYP(A-B)* group, a reference Mi.V concentrate DNA sample was used as positive control. For *GYP(A-B-A)* group, normal *GPA* served as the positive control. (Most *GYP(A-B-A)* hybrids are resulted from gene conversion with a small segment of *GYPB* between the junction of intron 2 and pseudoexon 3 replacing the homologous segment of *GYPA*. And, both primers are located in *GYPA*.) For *GYP(B-A-B)* and *GYP(B-A)* groups, we used previously collected and sequence-confirmed Mi.III and St^a^ blood samples as positive control, respectively. All PCR products were purified by using QIAquick PCR Purification Kit (Valencia, CA, USA). Then, the purified products were sequenced at the Sequencing Center of Chang Gung Memorial Hospital.

**Table 1:** PCR targets and their presumptive product length.

*GYP* variants	Primers		Targets	Nucleotides
*GYP(A-B)*	Forward	797A	MiV<$>\raster(50%)="rg2"<$>MiXI	1225
	Reverse	2026B		
*GYP(A-B-A)*	Forward	797A	MiI<$>\raster(50%)="rg2"<$>MiII<$>\raster(50%)="rg2"<$>MiVII	253
	Reverse	GPABA-E3R	MiVIII<$>\raster(50%)="rg2"<$>MiIX	
*GYP(B-A)*	Forward	797B	St^a^	1075
	Reverse	Sta-E4F		
*GYP(B-A-B)*	Forward	F2	MiIII<$>\raster(50%)="rg2"<$>MiIV	148
	Reverse	Rccgg	MiVI<$>\raster(50%)="rg2"<$>MiX	

**Table 2:** PCR primers used in this study, including their precise locations.

Primers used in this study			
Name of primer	Location	Sequence	Comment
797A*	nt 660-679 of IN2	5'-GCA GTC ACC TCA TTC TTG AC-3'	*GYPA*-specific, foward
2026B*	nt 116-87 of IN4	5'-GTT AAC AAC ATA TGC TCT TCT GTT TTA AG-3'	*GYPB*-specific, reverse
GPABA_E3R**	nt 110-80 of IN3	5'-ATG GGT TTT CTG TCA CGA AAG CTT GAG AAC T-3'	*GYPA*-specific, reverse
797B*	nt 663-682 of IN2	5'-GCA GTC ACC TCA TTC TTG TT-3'	*GYPB*-specific, forward
Sta_E4F**	nt 38-19 of EX4	5'-TGG TTC AGA GAA ATG ATG GG-3'	*GYPA*-specific, reverse
F2***	nt 683 of IN2 - nt 4 of EX3	5'-CCC TTT CTC AAC TTC TCT TAT ATG CAG ATA A-3'	*GYPB*-specific, forward
Rccgg***	nt 28 of IN3 - nt 91 of EX3	5'-GAG CAA CTA TTT AAA ACT AAG AAC ATA CCG G-3'	*GYPB*-specific, reverse

*From Ref. [7].

**Originally designed.

***From Ref. [13]. IN:intron. EX:exon.

**Table 3:** PCR programs used in this study.

*GYP* variants	Denaturation	Annealing	Extension	Cycles
*GYP(A-B)*	95°C 2 mins	60°C 2 mins	72°C 3 mins	35
*GYP(A-B-A)*	95°C 2 mins	60°C 2 mins	72°C 3 mins	35
*GYP(B-A)*	95°C 2 mins	58°C 2 mins	72°C 3 mins	35
*GYP(B-A-B)*	95°C 30 sec	58°C 30 sec	72°C 2 mins	30

### The Validation of Efficacy of Current Anti-‘Mi^a^’ Screening Method

Three hundred and eighty-nine (389) randomly collected patient's blood samples were tested with anti-‘Mi^a^’ sera obtained routinely in our blood bank.

Manual polybrene (MP) method without a supplementary anti-human globulin (AHG) phase was used for serologic testing, as our routine practice. Briefly, RBCs are incubated with the anti-‘Mi^a^’ sera in a low ionic medium at room temperature for one minute. Polybrene, a quaternary ammonium polymer, is then introduced to cause nonspecific red blood cell aggregation. The test tubes are centrifuged, the cell free supernatant fluid decanted, and the Polybrene effect on the cells is neutralized by adding a dilute sodium citrate-glucose solution. The entire procedure is completed in less than three minutes. The hemagglutination results are evaluated macroscopically and microscopically. Reaction intensity 1+ or more is defined as a positive result. Each blood sample was tested by anti-‘Mi^a^’ of five different individuals.

Genotypes of these tested blood samples were determined by the method mentioned above. The results of serologic test were then compared with genotypic results from PCR-sequencing data. The sensitivity and specificity of the serologic test were then calculated.

### The Significance of St^a^ Antigen in Taiwan Transfusion Practice

St^a^(+) genotyped RBCs, as tested in the previous section, were preserved for later use and were included in our blood bank for screening of anti-St^a^. Five thousand four hundred and thirty-one (5431) routine samples were tested. MP method with a supplementary AHG phase was used for the serologic test.

## Results

### The Frequencies of Glycophorin Variants Including Miltenberger Subtypes and St^a^

**Figure 1:**
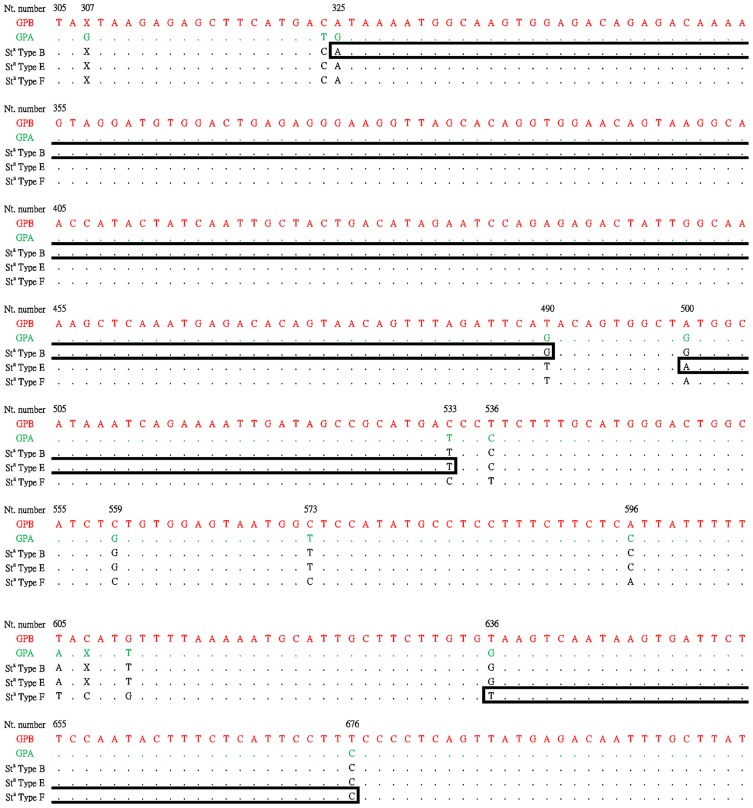
Comparison of the nucleotide sequences spanning genomic regions that contain the crossing-over sites in Intron 3 of the three types of St^a^ alleles. E and F are newly identified types in this study. Numbering of the nucleotides starts at the first nucleotide of the Intron 3. The locations of each recombination site of St^a^ variants are boxed. Positions of the nucleotides that are different in ordinary glycophorin genes and the variants are indicated.

GP.Mur (Mil.III) was confirmed in 57 of 1027 (5.6%) DNA sequenced samples. No other Miltenberger subtypes were found. For St^a^ group, DNA products were successfully amplified from 35 out of 1027 samples (3.4%). By sequencing, 29 of them belonged to the previous reported St^a^ type B, and the other 6 samples belonged to currently unreported St^a^ types. 4 of the 6 have the cross-over point within intron 3, between nucleotides 500 and 533 (we propose it to be called type E). The other 2 have the cross-over point between nucleotides 636 and 676 (we propose it to be called type F). For the comparison of nucleotide sequences, please refer to Figure 1.

### The Validation of Efficacy of Current Anti-‘Mi^a^’ Screening Method

By PCR and sequencing, 25 out of the 389 tested blood samples were proved to be Mil.III (GP.Mur) genotype. The prevalence of the Mil.III (GP.Mur) genotype is 6.4% (25/389). Each blood sample was subsequently tested by anti-‘Mi^a^’ of five different individuals. Among the 125 reactions carried out from 25 genetically GP. Mur(+) samples, 84 reactions were positive (sensitivity = 67.2%). 1694 negative results were found from 1820 reactions carried out by genetically GP.Mur(-) samples, and the specificity is 93.1% (1694/1820). Among 1945 serological reactions carried out from all 389 samples, 210 results were positive. Only 84 of these 210 positive results came from genetically GP.Mur(+) samples, and the positive predictive value is 40% (84/210). (Table 4)

**Table 4:** 2×2 table of the serology tests results.

	Genetically (+)	Genetically (−)
Serologically (+)	84	126
Serologically (−)	41	1694

### The Significance of St^a^ Antigen in Taiwan Transfusion Practice

Testing 5431 samples for the presence of anti-St^a^ using St^a^(+) genotyped RBCs, 7 antisera were found and the prevalence was about 0.13%. The strength of the reactions except one 3+ case are almost always weak (1+) in MP phase, and all of them included the 3+ case are negative when moving on AHG phase.

## Discussion and Conclusions

Screening for anti-‘Mi^a^’ has been incorporated in Taiwan transfusion practice for more than 20 years. The use of ‘Mi^a^’(+) screening RBCs for this purpose is based on the assumption that GP.Mur is the only glycophorin variant reacting with anti-‘Mi^a^’ in Taiwan. However, reports of serologic data and genetic data were conflicting [3], [7], [14]. Furthermore, the efficacy of this screening method had never been validated.

In this study, in accordance with two of the previous reports [3], [14], but against the other [7], we found no other Miltenberger subtypes except GP.Mur in Taiwan. This finding warrants the validity of using ‘Mi^a^’(+) screening RBCs to detect anti-‘Mi^a^’ in current practice. However, our study also showed that this screening method has a low sensitivity of 67.2% (84/125) and positive predictive value of 40% (84/210). The result was fairly consistent with a previous observation. Shih et al. found 19 serologically GP.Mur positive individuals out of 200 samples (9.5%) tested with anti-‘Mia’ sera [7], and we found 210 positive results out of 1945 serological reactions (10.8%). In addition, they found only 9 out of 19 (47.4%) serologically GP.Mur positive individuals were genetically GP.Mur positive, compared with 84 out 210 (40%) by us. Because of the low sensitivity, as much as 32.8% of those individuals with anti-‘Mi^a^’ may not be detected on antibody screening and are on the risk of receiving ‘Mi^a^’(+) transfusion. This problem can usually be avoided by pretransfusion major crossmatch, which is routinely applied in Taiwan transfusion practice. However, for institution utilizing computer crossmatch as pretransfusion incompatibility test, the false negative on anti-‘Mi^a^’ screening may result in clinically significant transfusion reactions.

Genotypic GP.Mur+ individuals can be identified by PCR-sequencing method from extracted genomic DNA as in this study, by direct blood PCR, or by high-resolution melting [14]. Theoretically, RBCs of these individuals will express glycophorin variant GP.Mur on the cell membrane, and these antigens will react with anti-GP.Mur (e.g anti-‘Mi^a^’ in Taiwan). Identifying these individuals could prevent transfusion of GP.Mur(+) RBCs to recipients with anti-GP.Mur. However, due to inconsistent expression of Miltenberger antigens, the serologic reaction can be weak or strong, ranging from non-existent, weak reaction requiring microscopic confirmation, to strong grade 3+ agglutination [14]. Although we do not know what will occur clinically when genotypically GP.Mur(+) but serologically nonreactive (to anti-GP.Mur) RBCs are transfused to an individual with anti-GP.Mur, the drastically higher sensitivity of genotyping could offer important safeguard.

The distribution of the St^a^ antigen, determined by serologic method using anti-St^a^, ranges from 0 to 5.2% among different populations of Taiwan [12]. Although three types of St^a^ were identified as early as in 1991 [15], few studies on St^a^ and no studies focused on the sensitivity or specificity of anti-St^a^ were carried out since then. We must emphasize the way we approach to St^a^ blood group is a reverse of the traditional serology-first (phenotype) strategy, but we consider this method is at least equivalent because of the characteristic of St^a^ blood group. The expression of St^a^ is due to the combination of exon 2 of *GYPB* and exon 4 of *GYPA*, resulted from gene crossing-over in intron 3. No matter where the alternation site in intron 3 is (resulting in different St^a^ genetic types), the genetic results on transcription and protein levels are the same. Thus, any St^a^ genetic types resulting from intron 3 crossing-over of *GYPB-A* will end up with the same peptide epitope, which is the so called St^a^ antigen, and should react with anti-St^a^. However, our study finds that the reactions are inconsistent and heterogeneous, ranging from no reaction at all to 3+ by MP method, and all of the MP+ cases did not react on AHG phase. We consider the so-called anti-St^a^ antiserum is non-specific and cold-agglutinated. Combined the low incidence (0.13%) and the cold-agglutination property of anti-St^a^, we consider routine screening of anti-St^a^ is not indicated, at least in Taiwan, without further evidence.

In conclusion, in this study we confirm the previous assumption that GP.Mur is the only prevalent Miltenberger subtype in Taiwan, and thus validate the current anti-‘Mia’ screening test used in Taiwan blood bank practice. The sensitivity of anti-‘Mia’ screening test is barely fair, but routine pretransfusion major crossmatch may prevent some of the cases and genotyping could act as a safeguard for those slipping through the net. Although the prevalence of St^a^ antigen in Taiwan is high, the incidence of anti-St^a^ is low, and the reactions are heterogeneous and probably cold-agglutination. Routine screening of anti-St^a^ in Taiwan is not necessary based on current evidence.
